# Cooking skills in undergraduates: Results of a multicenter Brazilian cross-sectional study

**DOI:** 10.1371/journal.pone.0345500

**Published:** 2026-04-30

**Authors:** José Douglas Bernardino Domingos, Manuela Mika Jomori, Erika Paula Silva Freitas, Eva Débora de Oliveira Andrade, Rafaela Nayara da Costa Pelonha, Ana Paula de Bulhões Vieira, Thaysa Barbosa Cavalcante Brandão, Bruna Merten Padilha, Thais Souza Passos, Bruna Leal Lima Maciel

**Affiliations:** 1 Graduate Program in Nutrition, Health Sciences Center, Federal University of Rio Grande do Norte, Natal, Rio Grande do Norte, Brazil; 2 Department of Nutrition, Federal University of Santa Catarina, Florianópolis, Santa Catarina, Brazil; 3 Graduate Program in Health Sciences, Health Sciences Center, Federal University of Rio Grande do Norte, Natal, Rio Grande do Norte, Brazil; 4 Faculty of Nutrition, Federal University of Alagoas, Alagoas, Brazil; International Medical University, MALAYSIA

## Abstract

Understanding university students’ cooking skills is essential for guiding actions aimed at improving dietary quality through culinary practices. The university environment offers a unique opportunity to promote health and quality of life among young adults, making the development of cooking-related competencies a valuable strategy for fostering healthy eating habits. This study aimed to analyze the cooking skills of undergraduate students. This is a cross-sectional study, with data collected between October 2020 and March 2021, with undergraduate students (n = 3138) from 4 public universities in Brazil. The Brazilian Questionnaire for the Assessment of Cooking Skills and Healthy Eating was used to collect data. Logistic regressions were used to assess the associations of cooking skills with socioeconomic variables. Of the total students, 72.6% were female, with a median age of 22 (20–26) years, and 68.0% demonstrated high cooking skills. High cooking skills were associated with female students, those who had learned how to cook through classes, courses, or school, as well as those who had acquired skills through self-directed methods, such as the internet, cookbooks, or TV programs.. Logistic regression analysis showed that across all universities, students with high knowledge of culinary terms and techniques and high availability and accessibility of fruits and vegetables were less likely to have low or intermediate culinary skills. Therefore, interventions focused on cooking skills in the study population should primarily target male students, encouraging undergraduates to learn how to cook independently, enhancing the availability and accessibility of fruits and vegetables, and increasing their knowledge of cooking terms and techniques.

## 1. Introduction

Cooking skills, according to Jomori et al. [1], are defined as “the confidence, attitude, and application of individual knowledge to perform cooking tasks, from menu planning and shopping to food preparation, whether natural, minimally processed, processed or ultra-processed foods.” The concept of cooking skills involves both task-centered skills, which include the use of different food and pre-preparation techniques, and person-centered skills, encompassing cooking knowledge, attitudes, confidence and behavior [1].

According to the Food Guide for the Brazilian Population [2], these skills are developed and perfected in each society and are transmitted over generations. The flavor, aroma, texture, and appearance that natural or minimally processed foods will acquire depend on them. Given this, studies indicate that the development of cooking skills promotes the adoption of healthy eating habits because when preparing food at home, the consumption of natural foods is favored, improving the quality of meals [2–4].

However, the dissemination of cooking skills has been decreasing over the generations, which has led young adults to show less confidence and independence in preparing their own meals [2]. The transition to college brings changes and challenges for young adults, such as leaving their parents’ home, developing autonomy and taking responsibility for meals’ preparation [5,6]. In addition, university students need to manage their time between academic demands and studies, and this scenario contributes to a greater propensity to consume ultra-processed foods, and convenience foods [7].

Consistent with this, Nelson et al. [8] pointed out changes in students’ eating habits, such as the decrease in the intake of fruits and vegetables and the increase in the consumption of fast food, snacks, and soft drinks, foods that require little or no cooking skills. This unhealthy behavior can have negative consequences for health, such as the emergence of chronic non-communicable diseases, such as diabetes, hypertension, and obesity [9]. Previous studies have assessed chronic noncommunicable diseases among university students [10–12]. Pelonha et al. [10] found that overweight and obesity were present in 29% and 15% of the university students, respectively, while Silva et al. [11] observed 25% and 5%, respectively. Hypertension and cardiovascular risks have also been investigated, with studies reporting up to 44% [12] of students showing increased cardiovascular risk, and 13% with elevated blood pressure, especially in males [12].

Cooking skills are directly associated with healthier eating patterns, characterized by a higher intake of natural and minimally processed foods [3,13–15].. Culinary interventions with university students have shown positive results, such as improved cooking skills [13,16–19], healthier food intake and/or better diet quality [14,17,19–22] reduced food insecurity, anxiety, and perceived stress [19]. Therefore, developing cooking skills in the university environment can be an effective strategy to improve the health of university students.

Ideally, interventions should be based on studies that characterize the target populations. Although some studies have already characterized culinary skills in university students, these have mostly been conducted with limited populations, considering students from only one university [7,23–26]. Overall, these studies have shown female students [4,26], those with higher age [24,26], studying in the health area courses [24] and having more time available for cooking [24] were associated with better cooking skills.

In Brazil, cooking skills are not traditionally taught in schools nor in the university curriculum. Borba et al. [7] found that Brazilian university students with low self-efficacy for using basic cooking techniques, self-efficacy for using fruits, vegetables, and seasonings and produce consumption self-efficacy were associated with having less than one hour a day to cook and not knowing how to cook. As far as we are concerned, no studies have been conducted to characterize cooking skills, considering different campuses in different regions of a country. Moreover, making an updated diagnosis with specific populations improves the targeting of interventions. Therefore, this study aimed to characterize the cooking skills of university students in Brazil. The study hypothesized that university students have low cooking skills.

## 2. Materials and methods

### 2.1. Ethical aspects

This research is a multicenter project entitled “Nutrition is in the kitchen! Cooking skills and healthy eating at university”, developed at the Federal University of Santa Catarina – UFSC (coordinating center), Federal University of Rio Grande do Sul – UFRGS, Federal University of Rio Grande do Norte – UFRN and Federal University of Alagoas – UFAL. The local projects were approved by their respective Human Ethics Committees (UFRN under CAAE 36572420.1.0000.5292, opinion 4.523.788, 02/09/2020; UFAL under CAAE: 09427219.5.3002.5013, opinion 4.171.141, 23/07/2020; UFSC under CAAE: 09427219.5.3001.0121, opinion 4.107.951, 24/06/2020; and UFRGS under CAAE: 09427219.5.1001.5347, opinion 4.047.329, 25/05/20). Each site only initiated local data collection after local ethics approvement. All volunteers registered their online written consent to participate in the study.

### 2.2. Type of study and study period

This was a cross-sectional study, with data collection between 25^th^ of June 2020 and 10^th^ of December 2022.

### 2.3. Data collection and population studied and

Data collection was conducted online via Google Forms platform. The online form contained sociodemographic characterization of the population and the Brazilian Questionnaire for the Assessment of Cooking Skills and Healthy Eating (QBHC). Non-probabilistic sampling was used. All undergraduates aged 18 or older at UFRN, UFAL, UFSC, and UFRGS enrolled in an undergraduate course were eligible for the present study and received an invitation to participate via institutional e-mail and the university’s social media. The invitation included a single link to the study’s consent form and research questions, which could be viewed only after completing the consent form. Those who agreed to participate through the informed consent form and responded to the study’s questions were included (n = 3348). From these, exclusions were applied based on being a graduate student, not completing the form correctly, and duplicate responses, resulting in 3138 participants: n = 785 at UFRN; n = 418 at UFAL; n = 1057 at UFSC; and n = 878 at UFRGS. Power was calculated *a posteriori* for *X*^2^ tests, using the GPower statistical software v. 3.1.9.7, and considering an effect size of 0.1, and an α of 5%, the calculated power for the final sample was 99%.

### 2.4. Sociodemographic characterization of the population

The Google Form presented questions about age, sex, race, course enrollment, living arrangement, family income, time available to cook per day, and how the student learned how to cook.

### 2.5. Cooking skills assessment

The Brazilian Questionnaire for the Assessment of Cooking Skills and Healthy Eating (QBHC) was used [27,28]. This questionnaire was developed and validated online among Brazilian undergraduates by Jomori et al. [29], based on the Cooking with Chef Program (CWC) questionnaire. The questionnaire has 7 scales, namely: 1) Availability and accessibility of fruits and vegetables; 2) Cooking attitude; 3) Cooking behavior; 4) Self-efficacy in fruits, vegetables, and greens consumption; 5) Self-efficacy in cooking; 6) Self-efficacy in using fruits, vegetables, and seasonings; and 7) Knowledge of cooking terms and techniques.

The scoring and classifications of the QBHC items were proposed through a validation study, utilizing a theoretical model [27,28]. Cooking skills were classified as low (20–43 points), intermediate (44–73 points), or high (74–100 points), considering the sum of the scores from the scales 2–6, as proposed by Jomori et al. [28]. The availability and accessibility of fruits and vegetables was classified as low (0–2 points), intermediate (3–6 points), or high (7–8 points) [27,29]. The knowledge of cooking terms and techniques was classified as high when the participant answered ≥6 items correctly and low when the participant answered <6 items correctly [27,29].

### 2.6. Data analysis

The data obtained through Google Forms were saved and coded in Microsoft Excel (2013) and analyzed in the Statistical Package for the Social Sciences (SPSS®) version 18.0 (IBM Corporation, Armonk, NY, 2011) [30].

For descriptive analysis of categorical variables, the distribution of absolute (N) and relative (%) frequencies was used. Continuous numeric variables were presented using mean (standard deviation) or median (Q1 – Q3), according to the normality of the data, examined by the Kolmogorov-Smirnov test. The Chi-square test was used to analyze whether there was a relationship between categorical variables, which included the analysis of sociodemographic variables by university studied and by cooking skills classification. The Kruskal-Wallis test was used to test the medians between more than two groups, and the Mann-Whitney test was used to test the medians between two groups, which were used when assessing the undergraduates’ age in the universities studied and the undergraduates’ age by cooking skills classification, respectively.

Logistic regression models were used to evaluate variables associated with low or intermediate cooking skills (low/intermediate cooking skills = 1; high cooking skills = 0) in each of the participating study centers. Four models were presented at the end, one for each center. The independent variables that showed a significant association with cooking skills in the bivariate analysis were initially placed individually in unadjusted models, with the unadjusted odds ratios (OR) and 95% confidence intervals (95% CI) being demonstrated (Supplementary table 1), to analyze the contribution of a single variable to low/intermediate cooking skills. Then, logistic regression models were calculated with all independent variables in the model.

The validation of the adjustment of the final models presented was ensured by observing the Omnibus test, in which p values were less than 0.05, and the Hosmer and Lemeshow test, in which p values were greater than 0.05. Thus, the final models incorporated the following independent variables: age, sex, income, if the student learned how to cook in a class, course, or school, if the student learned how to cook alone, on the internet, with a recipe book or TV program, time available to cook, availability and accessibility of fruits and vegetables and knowledge of cooking terms and techniques. Adjusted odds ratios (AORs) and their 95% confidence intervals were presented.

The p-value of 0.05 was adopted for the analysis performed in the study, when analyzing each university studied (Tables 1 and 3). However, for analysis in which the total sample size exceeded 1000 (Table 2), the p-value was deemed significant when less than 0.01 to avoid Type 1 errors.

**Table 1:** Characterization of the studied undergraduates (n = 3138).

Variables	Total	UFRN	UFAL	UFSC	UFRGS	p-value
		(n = 785)	(n = 418)	(n = 1057)	(n = 878)	
**Age, median (Q1 - Q3)**	22.0 (20.0–26.0)	23.0 (21.0–30.0)	22.0 (20.0–25.0)	22.0 (20.0–24.0)	22.0 (20.0–26.0)	0.000^1^
**Sex, n (%)**						
Male	860 (27.4)	234 (29.8)	108 (25.8)	284 (26.9)	234 (26.7)	0.362^2^
Female	2278 (72.6)	551 (70.2)	310 (74.2)	773 (73.1)	644 (73.3)	
Total	3138 (100.0)	785 (100.0)	418 (100.0)	1057 (100.0)	878 (100.0)	
**Race, n (%)**						
White	2155 (68.7)	414 (52.9)	153 (36.6)	867 (82.0)	721 (82.1)	0.000^2^
Yellow	32 (1.0)	6 (0.8)	8 (1.9)	12 (1.1)	6 (0.7)	
Black/brown	927 (29.6)	359 (45.8)	252 (60.3)	169 (16.0)	147 (16.7)	
Indigenous	8 (0.3)	3 (0.4)	1 (0.2)	4 (0.4)	0 (0.0)	
Did not inform	14 (0.4)	1 (0.1)	4 (1.0)	5 (0.5)	4 (0.5)	
Total	3136 (100.0)	783 (100.0)	418 (100.0)	1057 (100.0)	878 (100.0)	
**Course by area, n (%)**						
Humanities	969 (30.9)	303 (38.7)	118 (28.2)	343 (32.5)	205 (23.4)	0.000^2^
Exact Sciences	1049 (33.5)	210 (26.8)	128 (30.6)	453 (42.9)	258 (29.5)	
Health/Life Sciences	1114 (35.6)	270 (34.5)	172 (41.1)	259 (24.5)	413 (47.1)	
Total	3132 (100.0)	783 (100.0)	418 (100.0)	1055 (100.0)	876 (100.0)	
**Who do you live with, n (%)**						
Alone	449 (14.3)	68 (8.7)	24 (5.7)	229 (21.7)	128 (14.6)	0.000^2^
With parents or guardians	1730 (55.1)	492 (62.7)	313 (74.9)	448 (42.4)	477 (54.3)	
With partner/child	484 (15.4)	164 (20.9)	56 (13.4)	130 (12.3)	134 (15.3)	
With colleagues and others	475 (15.1)	61 (7.8)	25 (6.0)	250 (23.7)	139 (15.8)	
Total	3138 (100,0)	785 (100,0)	418 (100,0)	1057 (100,0)	878 (100,0)	
**Having children, n (%)**						
Yes	190 (6,1)	90 (11,5)	31 (7,4)	28 (2,6)	41 (4.7)	0.000^2^
No	2948 (93,9)	695 (88,5)	387 (92,6)	1029 (97,4)	837 (95.3)	
Total	3138 (100,0)	785 (100,0)	418 (100,0)	1057 (100,0)	878 (100.0)	
**Family income, n (%)**						
≤ 1.5 minimum wages	447 (14.3)	50 (6.4)	87 (20.8)	194 (18.4)	116 (13.2)	0.000^2^
> 1.5 minimum wages	2688 (85.7)	732 (93.6)	331 (79.2)	863 (81.6)	762 (86.8)	
Total	3135 (100.0)	782 (100.0)	418 (100.0)	1057 (100.0)	878 (100.0)	
**Time available for cooking/day, n (%)**						
<2h	792 (26.6)	198 (25.8)	83 (19.9)	265 (27.2)	246 (29.9)	0.002^2^
≥2h	2190 (73.4)	570 (74.2)	335 (80.1)	708 (72.8)	577 (70.1)	
Total	2982 (100.0)	768 (100.0)	418 (100.0)	973 (100.0)	823 (100.0)	
**Learned how to cook through a class, course or school, n (%)**						
Yes	275 (9.4)	87 (12.0)	22 (5.9)	81 (8.1)	85 (10.1)	0.003^2^
No	2660 (90.6)	635 (88.0)	350 (94.1)	915 (91.9)	760 (89.9)	
Total	2935 (100.0	722 (100.0)	372 (100.0)	996 (100.0)	845 (100.0)	
**Learned how to cook by themselves, on the internet, with a recipe book or TV show, n (%)**						
Yes	2105 (71.7)	597 (82.7)	224 (60.1)	691 (69.3)	593 (70.2)	0.000^2^
No	832 (28.3)	125 (17.3)	149 (39.9)	306 (30.7)	252 (29.8)	
Total	2937 (100.0)	722 (100.0)	373 (100.0)	997 (100.0)	845 (100.0)	
**Learned how to cook from mom, dad, grandma or other family members, n (%)**						
Yes	2519 (85.6)	622 (85.0)	311 (83.6)	856 (85.9)	730 (86.5)	0.558^2^
No	425 (14.4)	110 (15.0)	61 (16.4)	140 (14.1)	114 (13.5)	
Total	2944 (100.0)	732 (100.0)	372 (100.0)	996 (100.0)	844 (100.0)	

^1^p value for the Kruskal-Wallis test, ^2^p value for the Chi-square test. Federal University of Rio Grande do Norte (UFRN), Federal University of Alagoas (UFAL), Federal University of Santa Catarina (UFSC), Federal University of Rio Grande do Sul (UFRGS). p value considered significant < 0.05.

**Table 2:** Distribution of the studied population according to characterization variables and cooking skills.

Variables	Cooking skills			
	Low/intermediate	High	Total	p-value
**Age, median (Q1 - Q3)**	22.0 (20.0–26.0)	22.0 (20.0–26.0)	22 (20–26)	0.980^1^
**Sex, n (%)**				
Male	309 (35.9)	551 (64.1)	860 (100.0)	0.004^2^
Female	695 (30.5)	1583 (69.5)	2278 (100.0)	
**Race, n (%)**				
White	685 (31.8)	1470 (68.2)	2155 (100.0)	0.156^2^
Yellow	4 (12.5)	28 (87.5)	32 (100.0)	
Black/brown	309 (33.3)	618 (66.7)	927 (100.0)	
Indigenous	2 (25.0)	6 (75.0)	8 (100.0)	
Did not inform	4 (28.6)	10 (71.4)	14 (100.0)	
**Course by area, n (%)**				
Humanities	331 (34.2)	638 (65.8)	969 (100.0)	0.043^2^
Exact Sciences	345 (32.9)	704 (67.1)	1049 (100.0)	
Health/Life Sciences	326 (29.3)	788 (70.7)	1114 (100.0)	
**Who do you live with, n (%)**				
Alone	148 (33.0)	301 (67.0)	449 (100.0)	0.018^2^
With parents or guardians	586 (33.9)	1144 (66.1)	1730(100.0)	
With partner/child	131 (27.1)	353 (72.9)	484 (100.0)	
With colleagues and others	139 (29.3)	336 (70.7)	475 (100.0)	
**Having children, n (%)**				
Yes	63 (33.2)	127 (66.8)	190 (100.0)	0.723^2^
No	941 (31.9)	2007 (68.1)	2948 (100.0)	
**Family income, n (%)**				
≤ 1.5 minimum wages	141 (31.5)	306 (68.5)	447 (100.0)	0.850^2^
> 1.5 minimum wages	860 (32.0)	1828 (68.0)	2688 (100.0)	
**Time available for cooking/day, n (%)**				
<2h	285 (36.0)	507 (64.0)	792 (100.0)	0.001^2^
≥2h	650 (29.7)	1540 (70.3)	2190 (100.0)	
Total	935 (31.4)	2047 (68.6)	2982 (100.0)	
**Learned how to cook through a class, course or school, n (%)**				
Yes	46 (16.7)	229 (83.3)	275 (100.0)	0.000^2^
No	794 (29.8)	1866 (70.2)	2660 (100.0)	
**Learned how to cook by themselves, on the internet, with a recipe book or TV show, n (%)**				
Yes	536 (25.5)	1569 (74.5)	2105 (100.0)	0.000^2^
No	305 (36.7)	527 (63.3)	832 (100.0)	
**Learned how to cook from mom, dad, grandma or other family members, n (%)**				
Yes	708 (28.1)	1811 (71.9)	2519 (100.0)	0.042^2^
No	140 (32.9)	285 (67.1)	425 (100.0)	

^1^p value for the Mann-Whitney test, ^2^p the Chi-square test. p value considered significant < 0.01.

**Table 3:** Logistic regressions for variables associated with low/intermediate cooking skills in each of the universities studied.

Independent variables	Low/intermediate cooking skills							
	UFRN		UFAL		UFSC		UFRGS	
	AOR (95% CI)	p-value	AOR (95% CI)	p-value	AOR (95% CI)	p-value	AOR (95% CI)	p-value
**Age**	1.00 (0.98–1.02)	0.947	0.98 (0.95–1.01)	0.318	0.96 (0.93–0.99)	0.012	0.96 (0.93–0.99)	0.013
**Sex**								
Female	–		–		–		–	
Male	0.89 (0.60–1.32)	0.583	1.39 (0.83–2.34)	0.204	1.07 (0.75–1.52)	0.702	1.17 (0.78–1.75)	0.443
**Income**								
**≤** 1.5 minimum wages	–		–		–		–	
**>** 1.5 minimum wages	1.48 (0.69–3.18)	0.305	1.22 (0.69–2.12)	0.485	1.02 (0.68–1.53)	0.905	1.64 (0.94–2.86)	0.076
**Time available for cooking/day, n (%)**								
<2h/day	–		–		–		–	
≥2h/day	0.88 (0.59–1.31)	0.546	1.20 (0.68–2.13)	0.517	0.76 (0.54–1.07)	0.126	0.81 (0.56–1.19)	0.301
**Learned how to cook through a class, course or school**								
Yes	–		–		–		–	
No	1.04 (0.59–1.84)	0.876	1.78 (0.57–5.53)	0.317	2.02 (0.93–4.42)	0.075	1.76 (0.86–3.59)	0.117
**Learned how to cook by themselves, on the internet, with a recipe book or TV show**								
Yes	–		–		–		–	
No	1.83 (1.17–2.84)	0.007	1.18 (0.74–1.88)	0.480	1.45 (1.05–2.01)	0.024	1.93 (1.33–2.80)	0.001
**Availability and accessibility of fruits and vegetables**								
Low	–		–		–		–	
Intermediate	0.89 (0.43–1.85)	0.768	0.52 (0.20–1.30)	0.164	0.49 (0.25–0.93)	0.031	0.70 (0.31–1.61)	0.410
High	0.33 (0.16–0.65)	0.001	0.25 (0.11–0.54)	0.001	0.17 (0.09–0.32)	0.000	0.36 (0.16–0.79)	0.011
**Knowledge of cooking terms and techniques**								
Low	–		–		–		–	
High	0.35 (0.24–0.50)	0.000	0.56 (0.35–0.89)	0.015	0.47 (0.34–0.64)	0.000	0.41 (0.28–0.58)	0.000

Dependent variable: Low/intermediate cooking skills. AOR: adjusted odds ratio considering all variables in the model. Federal University of Rio Grande do Norte (UFRN), Federal University of Alagoas (UFAL), Federal University of Santa Catarina (UFSC), Federal University of Rio Grande do Sul. p value considered significant < 0.05. Unadjusted logistic regressions in each university studied for variables associated with low/intermediate cooking skills can be found in Supplementary table 1.

## 3. Results

### 3.1. Characterization of the studied population

The median age was 22.0 (20.0–26.0) years, with a higher age of 23.0 (21.0–30.0) years for participants from UFRN (Kruskal-Wallis test, p = 0.000). Females accounted for the largest proportion of participants, 72.6% of the total. Most participants (68.7%) identified themselves as white, followed by black/brown (29.6%), except for the UFAL students, where 60.3% identified themselves as black/brown (Chi-square, p = 0.000). Although the students were homogeneous in terms of their undergraduate courses in the three major areas, there was a higher percentage of students in the areas of health/life sciences at UFRGS (47.1%) and exact sciences at UFSC (42.9%) (Chi-square, p = 0.000). Most participants reported living with their parents or guardians (55.1%) (Chi-square, p = 0.000) and not having children (93.9%) (Chi-square, p = 0.000). Most students reported having a family income of more than 1.5 minimum wages (85.7%) (Chi-square, p = 0.000) and spending 2 hours or more a day cooking (73.4%, p = 0.002). In addition, 90.6% of the interviewees reported that they had not learned how to cook from a course or school (Chi-square, p = 0.003) and 71.7% reported that they had learned how to cook by themselves, on the internet, from a recipe book or TV program (Chi-square, p = 0.000). (Table 1).

### 3.2. Characterization of cooking skills

**Fig 1:**
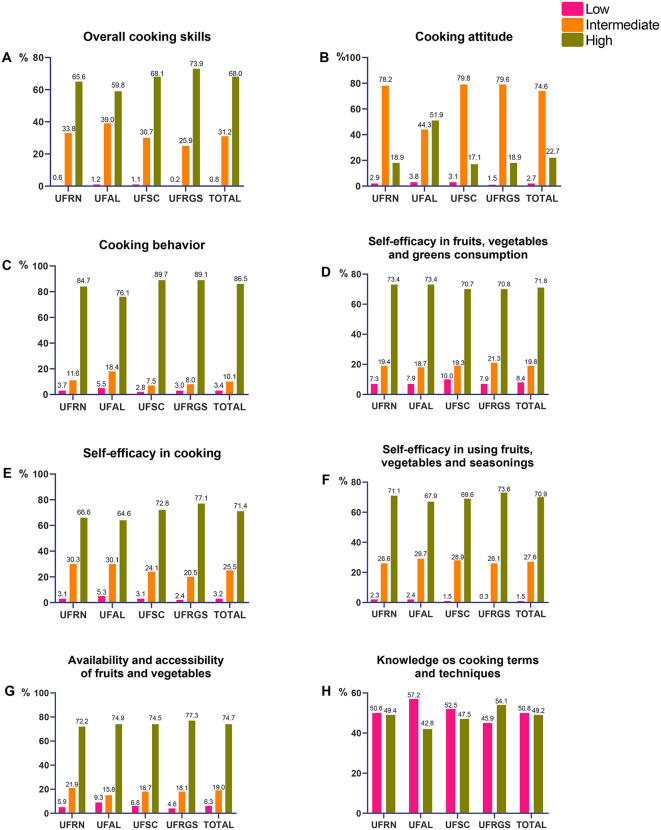
Cooking skills of the undergraduate students studied (n = 3138) by centers, according to the Brazilian Questionnaire for the Assessment of Cooking skills and Healthy Eating (QCSH). **(A)** Overall classification of cooking skills (Chi-square, p = 0.000); **(B)** Cooking attitude (Chi-square, p = 0.000); **(C)** Cooking behavior (Chi-square, p = 0.000); **(D)** Self-efficacy in fruits, vegetables, and greens consumption (Chi-square, p = 0.307); **(E)** Self-efficacy in cooking (Chi-square, p = 0.000); **(F)** Self-efficacy for using fruits, vegetables, and seasonings (Chi-square, p = 0.008); **(G)** Availability and accessibility of fruits and vegetables (Chi-square, p = 0.005); **(H)** Knowledge of cooking terms and techniques (Chi-square, p = 0.001).

Overall, most undergraduates showed favorable results in cooking skills and in the evaluated scales, with 68.0% of the undergraduates presenting high cooking skills (Fig 1). UFRGS consistently presented the highest frequencies of students with high levels of cooking skills (73.9%) (Fig 1A), cooking behavior (89.7%) (Fig 1C), self-efficacy in cooking (77.1%) (Fig 1E), self-efficacy for using fruits, vegetables, and seasonings (73.6%) (Fig 1F), availability and accessibility of fruits and vegetables (77.3%) (Fig 1G), and knowledge of cooking terms and techniques (54.1%). UFSC showed the highest proportion of students with high cooking behavior (89.7%) (Fig 1H). In contrast, UFAL undergraduates generally had the lowest scores, with the highest frequencies of low cooking skills (1.2%) (Fig 1A), low cooking attitude (3.8%) (Fig 1B), low cooking behavior (5.5%) (Fig 1C), low self-efficacy in cooking (5.3%) (Fig 1E), and low availability and accessibility of fruits and vegetables (9.3%) (Fig 1G). The Chi-square test indicated significant associations (p < 0.05) for most variables, except for self-efficacy in consuming fruits, vegetables, and greens (p = 0.307).

### 3.3. Cooking skills according to sociodemographic characteristics

Because overall only 0.6% of the undergraduates presented low cooking skills, low and intermediate cooking skills were combined to assess variables associated with the culinary skills levels (Table 2). More females (69.5%) presented high cooking skills among the students (Chi-square, p = 0.004), and among those who reported having ≥ 2 hours per day available for cooking, 70.3% presented high cooking skills (Chi-square, p = 0.001). High cooking skills were also more frequent among those who reported having learned how to cook from a class, course, or school (83.3%) (Chi-square, p = 0.000) and among those who reported having learned how to cook by themselves, on the internet, from recipe books or TV programs (74.5%) (Chi-square, p = 0.000) (Table 2).

### 3.4. Logistic regressions for variables associated with low or intermediate cooking skills

Because cooking skills varied significantly across universities, we examined the characteristics associated with cooking skills at each of the universities studied. Unadjusted logistic regressions in each university studied for variables associated with low/intermediate cooking skills can be found in Supplementary table 1. According to the adjusted logistic regressions (Table 3), age was associated with low/intermediate cooking skills, with each additional year of age reducing the chances of having low/intermediate cooking skills by 4% in students from UFSC (AOR = 0.96; 95% CI = 0.93–0.99) and UFRGS (AOR = 0.96; 95% CI = 0.93–0.99). Participants who reported not having learned how to cook by themselves, on the internet, from a recipe book or TV program had higher odds ratios for low/intermediate cooking skills at UFRN (AOR = 1.83; 95% CI = 1.17–2.84), UFSC (AOR = 1.45; 95% CI = 1.05–2.01) and UFRGS (AOR = 1.93; 95% CI = 1.33–2.80). Students with high availability and accessibility of fruits and vegetables presented lower chances for low/intermediate cooking skills in all the universities studied: UFRN (AOR = 0.33; 95% CI = 0.16–0.65), UFAL (AOR = 0.25; 95% CI = 0.11–0.54), UFSC (AOR = 0.17; 95% CI = 0.09–0.32) and UFRGS (AOR = 0.36; 95% CI = 0.16–0.79). The intermediate availability and accessibility of fruits and vegetables also reduced the chances of low/intermediate cooking skills, but only for students from UFSC (AOR = 0.49; 95% CI = 0.25–0.93). The odds ratios for having low/intermediate cooking skills were also reduced by high knowledge of cooking terms and techniques in students from all the studied universities: UFRN (AOR = 0.35; 95% CI = 0.24–0.50), UFAL (AOR = 0.56; 95% CI = 0.35–0.89), UFSC (AOR = 0.47; 95% CI = 0.34–0.64) and UFRGS (AOR = 0.41; 95% CI = 0.28–0.58) (Table 3).

## 4. Discussion

This study characterized and analyzed the cooking skills of university students in Brazil. The results of the bivariate analysis showed that being female and having learned how to cook in a class, course, or school or through self-directed methods, such as the internet, cookbooks, or watching TV programs, were related to high cooking skills. In addition, the logistic regressions showed that age, having intermediate or high availability and accessibility of fruit and vegetables, and high knowledge of cooking terms and techniques decreased the likelihood of having low or intermediate cooking skills. On the other hand, students who did not learn how to cook by themselves, with the help of the internet, recipe books, or TV programs, had higher odds of having low or intermediate cooking skills.

Although the initial hypothesis of the study was refuted, as most of the students had high cooking skills, the results obtained are still relevant, allowing for the observation of characteristics associated with low or intermediate cooking skills, which should be considered when targeting interventions to promote healthy eating through cooking skills among the studied population.

A higher proportion of female participants demonstrated high cooking skills in this study, consistent with previous findings showing greater cooking confidence and self-efficacy among women [26,31]. Women are often more involved in domestic cooking activities [32,33], a pattern linked to traditional gender roles and societal expectations [34], which may partly explain our results.

Another finding in the present study was that high cooking skills were related to having learned how to cook from a class, course, or school, as well as independently from the internet, recipe books, or TV programs. This result may be explained by the fact that individuals with an interest in cooking actively seek opportunities to learn, which contributes to the development of higher cooking skills. Such interest may encourage students to invest effort in acquiring culinary knowledge through online resources, social media, or by observing others. This proactive behavior can enhance both their self-efficacy and practical cooking skills. A study [15] with university students at Western University in London showed that people who took part in a cooking food and nutrition course had higher cooking skills compared to those who did not. In addition, those who underwent intervention programs acquired or improved their cooking skills. In a study with Brazilian university students, Souza & Kotzias [35] found that most participants learned how to cook on their own or with the help of the internet. This may be due to the ease with which the internet enables learning new things on various subjects [36].

Intending to evaluate whether an experimental cooking course could improve the cooking and eating skills, vegetable and fruit consumption, and other eating behaviors of high school students from the French-speaking school district in New Brunswick, Canada, LeBlanc et al. [37] identified that cooking and eating skills increased in the intervention group when compared to the control group and that before the intervention the skills were few.

Our results also indicate that stimulating undergraduates to learn how to cook independently through readily available resources, such as the Internet, may be a strategy to contribute to healthier eating and health promotion. This was reinforced by the fact that for the variable “learned how to cook on their own, from the internet, cookbooks or TV programs,” when the logistic regression models were run, participants who did not learn to cook from these sources showed higher odds ratios of having low/intermediate cooking skills. This result could be explained by a lack of interest on the part of the students in exploring the means of learning cooking skills or by seeking other sources, such as cooking classes and observing other family members or friends.

In the study by Souza & Kotzias [35], conducted at the Federal University of Santa Catarina in Brazil, a similar result was observed, affirming the importance of these sources in learning, with the mother, the internet, and self-learning being the primary sources for learning how to cook. The authors also attribute this fact to the SARS-CoV-2 coronavirus (COVID-19) pandemic, as the study was conducted during this period when people were isolated at home with their families and consequently learned to cook from their mothers, the internet, and on their own.

In this study, most participants (68.0%) demonstrated high cooking skills, intermediate cooking attitude (74.6%), high cooking behavior (86.5%), high self-efficacy in fruits, vegetables, and greens consumption (71.8%), high self-efficacy in cooking (71.4%) and high self-efficacy for using fruits, vegetables, and seasonings (70.9%). For the scales not considered when calculating cooking skills, we found that the majority had high availability and accessibility of fruits and vegetables (74.7%) and low knowledge of cooking terms and techniques (50.8%). Interestingly, in UFRGS there was a higher frequency of students with high cooking skills. This result could be attributed to the fact that UFRGS had a higher number of students in the health area, but the tested regression models did not corroborate this.

The logistic regressions also showed that students with intermediate availability and accessibility of fruits and vegetables (AOR = 0.49; 95% CI = 0.25–0.93) from UFSC, as well as students with high availability and accessibility of fruits and vegetables from all the universities studied, had lower odds of presenting low or intermediate cooking skills. This association is consistent with findings from intervention studies using the QBHC. In the randomized trial by Bernardo et al. [13], a six-week cooking intervention with Brazilian university students, including practical cooking classes and sessions on food selection and purchasing, led to greater availability and accessibility of fruits and vegetables, as well as improved self-efficacy and cooking attitudes. Similarly, Dezanetti et al. [38] found that most participants showed high availability and accessibility of fruits and vegetables (73.0%) and high cooking skills (70.7%), supporting the link between these variables. Individuals with better access to fresh foods are more likely to prepare meals from scratch, which enhances cooking skills. Moreover, the pleasure derived from cooking may further reinforce these behaviors, promoting higher fruit and vegetable intake and reducing the consumption of ready-made and industrialized foods.

Across all universities studied, higher knowledge of cooking terms and techniques was associated with lower odds of low or intermediate cooking skills. This result aligns with previous intervention studies, which have shown that cooking programs improve students’ overall cooking skills, technical knowledge, and self-efficacy in cooking [39]. These findings indicate that technical knowledge and confidence are key factors influencing cooking performance, diet quality, and the use of basic ingredients in meal preparation [1].

Therefore, we emphasize the importance of developing studies that address the variables highlighted in the results, enabling the development of strategies targeting variables associated with improved cooking skills.

This study has some limitations. The study included undergraduates from universities across different regions of Brazil, with a large number of participants. However, because the sampling was non-probabilistic and based on voluntary participation, the results cannot be generalized to all Brazilian undergraduate students. Another limitation was related to the higher percentage of female participants, which can be explained by the fact that topics like this generate more interest from this audience [40]. This fact is one of the reasons sex was used as an adjustment variable in the regression models. Nevertheless, the cited limitations do not invalidate the associations found. Moreover, ideally, cooking skills assessment should consider direct assessment of cooking skills, which is not possible with the available instruments, as most evaluate self-perceived cooking skills, especially person-centered skills. Thus, further studies should focus on developing more robust tools to capture all dimensions of culinary competence [41].

One of the study’s strengths was the relatively large number of participants, which allowed the examination of several variables through multivariable regression models, even though the non-probabilistic design limits generalizability. Another strength was the use of a cooking skills assessment questionnaire validated for online use in Brazilian university students [42]. Thus, our results contribute significantly to the literature on studying cooking skills among university students, which can serve as an important target for improving healthy eating [14,17,19–21]. In addition, the present study can be used to ground future research, cooking interventions, and extension projects aimed at undergraduates and for the academic community to promote the dissemination of cooking skills since they are directly related to eating habits and lifestyle, directly impacting health [43,44].

## 5. Conclusion

Most undergraduates presented high cooking skills. Being female, of higher age, with high availability and accessibility of fruits and vegetables, and possessing a high knowledge of cooking terms and techniques were inversely associated with low or intermediate cooking skills. However, not having learned how to cook alone, on the internet, with a cookbook, or through TV programs increased the chances of having low or intermediate cooking skills. Therefore, interventions focused on cooking skills need to be aimed mainly at male students, not only encouraging the participation of this gender but also serving as a stimulus for people to learn how to cook alone, improve the availability and accessibility of fruits and vegetables, and knowledge of cooking terms and techniques, stimulating all sources that contribute to knowledge and learning. Thus, the university environment emerges as an ideal space to promote health through the development of cooking skills. The findings may inform universities, health educators, and policymakers in designing evidence-based strategies to enhance cooking skills among students. These may include integrating hands-on culinary education into undergraduate curricula, offering extracurricular workshops or community-based cooking programs, and implementing public policies that expand access to healthy food preparation resources and facilities on campuses.

## Supporting information

S1 Table Unadjusted logistic regressions in each university studied for variables associated with low/intermediate cooking skills. (DOCX)
