# Supplementary material for: Cooking skills in undergraduates: Results of a multicenter Brazilian cross-sectional study
Source: PLoS One. 2026 Apr 30;21(4):e0345500. doi: 10.1371/journal.pone.0345500 (PMC13132208; doi:10.1371/journal.pone.0345500)
Supplement: S1 Table — (DOCX) [file pone.0345500.s001.docx]

**Supplementary table 1.** Unadjusted logistic regressions in each university studied for variables associated with low/intermediate cooking skills.

| **Independent variables** |  | **Low/intermediate cooking skills** | | | | | | | | | |  |  |
| --- | --- | --- | --- | --- | --- | --- | --- | --- | --- | --- | --- | --- | --- |
|  | **UFRN** | | | | **UFAL** | | **UFSC** | | **UFRGS** | |  |  |  |
|  | **OR (95% CI)** | | **p-value** | **OR (95% CI)** | | **p-value** | **OR (95% CI)** | **p-value** | **OR (95% CI)** | **p-value** |  |  |  |
| **Age** | 0.99 (0.97 - 1.01) | | 0.671 | 0.99 (0.97 - 1.02) | | 0.910 | 0.97 (0.95 - 0.99) | 0.024 | 0.95 (0.93 - 0.98) | 0.002 |  |  |  |
| **Sex** | | | | | | | | | | | |  |  |
| Female | - | |  | - | |  | - |  | - |  |  |  |  |
| Male | 1.19 (0.86 - 1.63) | | 0.285 | 1.71 (1.10 - 2.67) | | 0.016 | 1.25 (0.94 - 1.67) | 0.120 | 1.19 (0.85 - 1.66 | 0.300 |  |  |  |
| **Income** | | | | | | | | | | | |  |  |
| **≤** 1.5 minimum wages | - | |  | - | |  | - |  | - |  |  |  |  |
| **>** 1.5 minimum wages | 1.35 (0.71 - 2.56) | | 0.345 | 1.06 (0.65 - 1.71) | | 0.812 | 0.83 (0.60 - 1.16) | 0.295 | 1.33 (0.83 - 2.13) | 0.234 |  |  |  |
| **Time available for cooking/day, n (%)** | | | | | | | | | | | |  |  |
| <2h/day | - | |  | - | |  | - |  | - |  |  |  |  |
| ≥2h/day | 0.72 (0.52 - 1.01) | | 0.063 | 1.08 (0.66 - 1.78) | | 0.734 | 0.60 (0.44 - 0.81) | 0.001 | 0.73 (0.52 - 1.03) | 0.075 |  |  |  |
| **Learned how to cook through a class, course or school** | | | | | | | | | | | |  |  |
| Yes | - | |  | - | |  | - |  | - |  |  |  |  |
| No | 1.49 (0.88 - 2.53 | | 0.134 | 2.62 (0.87 - 7.93) | | 0.087 | 3.13 (1.59 - 6.16) | 0.001 | 2.01 (1.07 - 3.78) | 0.030 |  |  |  |
| **Learned how to cook by themselves, on the internet, with a recipe book or TV show** | | | | | | | | | | | |  |  |
| Yes | - | |  | - | |  | - |  | - |  |  |  |  |
| No | 2.12 (1.43 - 3.16) | | 0.000 | 1.39 (0.90 - 2.14) | | 0.130 | 1.51 (1.13 - 2.02) | 0.005 | 1.92 (1.37 - 2.67) | 0.000 |  |  |  |
| **Availability and accessibility of fruits and vegetables** | | | | | | | | | | | |  |  |
| Low | - | |  | - | |  | - |  | - |  |  |  |  |
| Intermediate | 0.82 (0.42 - 1.58) | | 0.565 | 0.44 (0.19 - 1.02) | | 0.57 | 0.62 (0.35 - 1.07) | 0.086 | 0.52 (0.25 - 1.04) | 0.067 |  |  |  |
| High | 0.28 (0.15 - 0.53) | | 0.000 | 0.23 (0.11 - 0.48 | | 0.000 | 0.24 (0.15 - 0.40) | 0.000 | 0.25 (1.13 - 0.49) | 0.000 |  |  |  |
|  |  |  |  |  |  |  |  |  |  |  |  | |  |
| **Knowledge of cooking terms and techniques** | | | | | | | | | | | |  | |
| Low | - | |  | - | |  | - |  | - |  |  | |  |
| High | 0.27 (0.20 - 0.37) | | 0.000 | 0.43 (0.28 - 0.65) | | 0.000 | 0.36 (0.27 - 0.47) | 0.000 | 0.31 (0.22 - 0.42) | 0.000 |  | |  |

Dependent variable: Low/intermediate cooking skills. OR: crude odds ratio from bivariate analysis
